# Recognition of Two Distinct Pathways for Trafficking of Proteins to the Apicoplast

**DOI:** 10.1128/mBio.02634-21

**Published:** 2021-12-21

**Authors:** Aparna Prasad, Swati Patankar

**Affiliations:** a Department of Biosciences & Bioengineering, Indian Institute of Technology Bombay, Powai, Mumbai, India; UT Southwestern Medical Center; University of Arizona

**Keywords:** *Toxoplasma gondii*, protein trafficking, vesicular trafficking

## LETTER

In their recent article (1), Cao and colleagues identify soluble *N*-ethylmaleimide-sensitive factor attachment protein receptor (SNARE) proteins that play roles in trafficking to the apicoplast of Toxoplasma gondii. As SNAREs are involved in the vesicular trafficking pathway, it is important to note that multiple routes exist for apicoplast trafficking in Toxoplasma gondii and Plasmodium falciparum. One route leads directly from the endoplasmic reticulum (ER) to the apicoplast (2, 3), and the other route leads from the ER to the apicoplast via the Golgi pathway (4, 5).

Cao and colleagues (1) elegantly perform conditional knockdown of several SNAREs and assay the effects of these experiments on the transport of proteins to the apicoplast using the marker proteins *T. gondii* ACP (TgACP), TgCPN60, TgATrx1, TgATrx2, TgFtsH1, and TgAPT1. In this section, the authors make a broad statement about the Golgi pathway being involved in the trafficking of apicoplast proteins. To substantiate this statement, they show that a conditional knockdown of TgGS27 and TgTrs85, SNAREs in the *trans*-Golgi network, leads to the disruption of protein transport to the apicoplast (Fig. 4 in reference 1). These data are interpreted to mean that the two SNAREs play roles in trafficking of the apicoplast marker proteins.

A broad statement about dependency on the Golgi pathway is surprising not only because it fails to acknowledge the substantial literature on Golgi pathway-independent trafficking of apicoplast proteins but also because in an article from our lab, we use an HDEL-mediated ER retention strategy to show that proteins localized to the apicoplast alone use the Golgi-independent trafficking route from the ER to the apicoplast (5). Of the apicoplast markers chosen by Cao and colleagues, TgACP (6), TgATrx2, and TgATrx1 (5) have been tested using the HDEL strategy and are unambiguously demonstrated to bypass the Golgi pathway. In contrast to proteins localized to the apicoplast alone, proteins that are targeted to both the apicoplast and the mitochondrion (TgTPx1/2, TgSOD2, or TgACN) use the Golgi-dependent trafficking route from the ER to the apicoplast (5). Mutant versions of these proteins that are targeted only to the apicoplast also use the Golgi pathway. Unfortunately, none of these dually targeted proteins have been tested by Cao and colleagues.

Instead, in order to test the involvement of the Golgi pathway in the transport of TgFtsH1, the authors used brefeldin A (BFA), a small molecule that can show off-target effects, both on the parasite and on host cells. Consistently with this, the TgFtsH1 signal from the parasites has completely disappeared in the presence of BFA (Fig. 7F in reference 1) when one expects to find it in the ER. Moreover, the lack of an apicoplast marker in this experiment makes it difficult to interpret the results. There is no direct evidence that TgAPT1 is transported through the Golgi-independent pathway.

As three out of the five marker proteins tested in the report reach the apicoplast via the Golgi-independent pathway, one wonders how knockdown of SNAREs that reside in the *trans*-Golgi network affect their transport. We argue that the phenotypes are pleiotropic rather than merely affecting apicoplast trafficking. Data in Fig. 5 in reference 1 show that TgGS27 and TgTrs85 affect the lipid metabolism of parasites, which can alter apicoplast structure, ranging from defects in morphology to a complete loss of the organelle (7). Therefore, the mis-localization of these apicoplast marker proteins may be due to the loss of the apicoplast itself and not due to dependency on the Golgi pathway.

Due to the concerns listed above, we strongly believe that Cao and colleagues have not tested any authentic Golgi-dependent apicoplast protein in their report and hence that their data might have alternate interpretations. We believe that the field of apicoplast protein trafficking would benefit enormously from a cognizance of the two pathways and the appropriate use of validated protein markers that utilize either of the pathways.
